# Fisetin ameliorates cognitive impairment by activating mitophagy and suppressing neuroinflammation in rats with sepsis‐associated encephalopathy

**DOI:** 10.1111/cns.13765

**Published:** 2021-11-27

**Authors:** Hongguang Ding, Ya Li, Shenglong Chen, Yin Wen, Shiying Zhang, Ensi Luo, Xusheng Li, Wenhong Zhong, Hongke Zeng

**Affiliations:** ^1^ Department of Emergency Medicine Guangdong Provincial People’s Hospital Guangdong Academy of Medical Sciences Guangzhou China; ^2^ Department of Emergency Medicine Beijing Key Laboratory of Cardiopulmonary Cerebral Resuscitation Beijing Chaoyang Hospital Capital Medical University Beijing China; ^3^ Department of Critical Care Medicine Guangdong Provincial People’s Hospital Guangdong Academy of Medical Sciences Guangzhou China; ^4^ Department of Endocrinology Binhaiwan Central Hospital of Dongguan Dongguan Hospital Affiliated to Medical College of Jinan University Dongguan China

**Keywords:** fisetin, mitophagy, neuroinflammation, NLRP3 inflammasome, sepsis‐associated encephalopathy

## Abstract

**Background:**

Fisetin, the effective ingredient of the traditional Chinese medicine named Cotinus coggygria, is recommended to be active therapeutic in many disorders. However, its role in sepsis‐associated encephalopathy (SAE) remains unclarified.

**Methods:**

Cecal ligation and puncture (CLP) operation was performed to establish a rat model of SAE. Rats were grouped according to the surgery operation and fisetin administration. Cognitive impairment was assessed by Morris water maze test. Disruption of blood‐brain barrier (BBB) integrity was detected by Evan's blue staining. The mitophagy, reactive oxygen species (ROS) generation, NLRP3 inflammasome activation, and pro‐inflammatory cytokines levels were measured through western blot and double immunofluorescence labeling. A transmission electron microscope was applied for the observation of mitochondrial autophagosomes.

**Results:**

Rats in the CLP group presented increased expression of IL‐1R1, pNF‐κB, TNF‐α, and iNOS in microglial cells, indicating severe inflammation in the central nervous system (CNS). Nevertheless, there was no increase in BBB permeability. Meanwhile, NLRP3 inflammasome was activated in cerebral microvascular endothelial cells (CMECs), presented with an elevation of caspase‐1 expression and IL‐1β secretion into CNS. In addition, we found fisetin significantly improved cognitive dysfunction in rats with SAE. Neuroprotective effects of fisetin might be associated with inhibition of neuroinflammation, represented with decreased expression of IL‐1R1, pNF‐κB, TNF‐α, and iNOS in microglia. Furthermore, fisetin induced mitophagy, scavenged ROS, blocked NLRP3 inflammasome activation of CMECs, as evidenced by decreased expression of caspase‐1 and reduced release of IL‐1β into CNS.

**Conclusion:**

Collectively, fisetin‐blocked NLRP3 inflammasome activation via promoting mitophagy in CMECs may suppress the secretion of IL‐1β into CNS, reduce neuroinflammation, and contribute to the amelioration of cognitive impairment.

## Graphical abstract

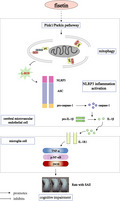

## 1 INTRODUCTION

Sepsis is one of the global healthcare problems that threatens the life of the public. It has been reported that the incidence of ICU‐treated sepsis cases was 58 per 100,000 person‐years, among which mortality reached 41.9%.^1^ Induced by the dysregulated inflammatory response to infection, sepsis contributes to multiorgan dysfunction. The central nervous system (CNS) is usually impacted at the early stage of sepsis and developed into sepsis‐associated encephalopathy (SAE), which accounts for 49.6% of all sepsis patients.^2^ SAE is featured by diffused cerebral dysfunctions, including delirium, hallucination, reduced concentration, changes in consciousness, and so on.^3^ Besides, SAE is responsible for increased mortality and long‐time sequelae. Based on considerable statistical analyses, the mortality rate of SAE was 13% higher than that of sepsis.^4^ However, there are limited therapeutic measures of SAE, including merely anti‐infective therapy and fundamental supportive treatments.^5^ In consideration of the high incidence and mortality, there is a driving unmet need to understand the physiopathologic mechanism of SAE and to develop new drugs for practical treatment.

Fisetin is a kind of bioactive flavanol that is ubiquitous in vegetables and fruits.^6^ Besides, fisetin is the effective ingredient of traditional Chinese medicine named Cotinus coggygria. Accumulating lines of evidence show multiple pharmacologic effects of fisetin encompassing anti‐cancer, anti‐angiogenesis, anti‐aging, and neuroprotection properties.^7^, ^8^, ^9^, ^10^ However, the underlying mechanism concerning neuroprotective effects of fisetin on SAE remains to be clarified.

In this study, we hypothesized that sepsis may induce the activation of NLR family pyrin domain containing 3 (NLRP3) inflammasome in cerebral microvascular endothelial cells (CMECs), increase secretion of Interleukin 1β (IL‐1β) into CNS, which can cause serious neuroinflammation and cognitive impairment. Fisetin can improve cognitive dysfunction in rats with SAE. It was surmised that fisetin may exert its neuroprotective effect through inducing mitophagy, blocking NLRP3 inflammasome activation of CMECs, suppressing the secretion of IL‐1β into CNS and inhibiting neuroinflammation.

## 2 MATERIALS AND METHODS

### 2.1 Animals and groups

Forty male rats (200–220 g, 6–8 weeks) were provided by South Medical University. All rats were randomly assigned into four groups (10 rats per group): cecal ligation and puncture group (CLP group), CLP + placebo group, CLP + fisetin group, and sham‐operated group (sham group). The former three groups underwent cecal ligation and puncture operation, while the sham group was only subjected to laparotomy. Rats in the CLP + fisetin group and CLP + placebo group were pretreated with intragastrical administration of fisetin (20 mg/kg) and an equal volume of the solvent of fisetin (0.5% DMSO solution) injection once a day for three consecutive days before CLP, respectively. All experiments were approved by the Research Ethics Committee of Guangdong Provincial People's Hospital (NO: GDREC2019836A). The animal data reporting of the current manuscript has followed the ARRIVE 2.0 guidelines.^11^

### 2.2 The rat model of cecal ligation and puncture

The CLP rat model was established by the cecal ligation and puncture operation in accordance with previous studies.^12^ Briefly, after intraperitoneal anesthetization with an injection of 3% pentobarbital sodium (30 mg/kg), the abdominal cavity was opened through a midline incision. Then, the cecum was carefully separated from peripheral tissue, ligated in the middle portion, and punctured 4 or 5 times with a sterile needle. A small amount of feces was squeezed out of the large intestine before repositioning of the cecum and closure of the abdomen.

### 2.3 Morris water maze test

The Morris water maze (MWM) test was conducted to reflect the learning and memory abilities of rats in different groups.^13^ The apparatus was composed of a circular pool with a diameter of 200 cm, a diameter cylinder 1 cm platform under the surface of the water, and a camera fixed over the pool which was used to track the behavior of rats. The pool was full of water (25 ± 1°C) and separated into four equal quadrants. After one‐day treatment in the pool, rats were educated to familiarize themselves with the specific circumstance. In the following tests, the time length to find the platform was taken by each rat to find the platform was recorded and defined as latency time. The frequency of crossing to the platform was also documented.

### 2.4 Evan's blue staining

Permeability of the blood‐brain barrier (BBB) was evaluated by measuring the extravasation of Evan's blue (EB) in the brain tissue as previously described.^14^ Briefly, rats were injected with 2% EB (4 ml/kg) via the tail vein 24 h after treatment. After deep anesthesia and quick sacrifice, the hearts of rats were perfused with continuous phosphate‐buffered saline (PBS) until the liquid from auricula dextran became clear. And 4% paraformaldehyde was then infused to fix the shape of the tissue. The brains were harvested and cut into slices along the coronal plane. Formamide (1 ml/100 mg) was added to incubate the slices at 60°C for 24 h. The concentration of EB extracted from each brain was determined at 620 nm using spectrophotometry.

### 2.5 ROS evaluation

A reactive oxygen species (ROS) enzyme‐linked immunosorbent assay (ELISA) kit (Dogesce; cat. no. DG21175D) was used to detect the level of ROS.^15^ Based on the manufacturer's instruction, the brain tissue samples were homogenized and then added into the special plate coated with antibodies that could capture ROS. After one‐hour incubation at 37°C, substrate A and B were added successively. Then, the plate was incubated in a darkroom at 37°C for about 20 min. Terminated by the stop buffer, the concentration of ROS in the tissue samples was measured with a spectrophotometer at 450 nm.

### 2.6 Western blotting analysis

Total proteins from the hippocampus tissue were extracted using a total proteins Extraction Kit (BestBio; cat. no. BB‐3101‐100T). The protein samples were firstly separated in a 10% SDS‐PAGE gel and then transferred to PVDF membranes. Blocked with 5% non‐fat milk, the membranes were incubated with the primary antibodies overnight at 4°C: Pink1 (1:1000, Abcam; cat. no. ab23707), Parkin (1:1000, Abcam; cat. no. ab233434), LC3B (1:1000, Abcam; cat. no. ab63817), SQSTM1/p62 (1:1000, Cell Signaling Technology; cat. no. 23214), caspase‐1 (1:1000, Abcam; cat. no. ab1872), IL‐1β (1:1000, Cell Signaling Technology; cat. no. 12703), IL‐1R1 (1:1000, Santa Cruz Biotechnology; cat. no. sc‐689), NF‐κB (1:1000, Cell Signaling Technology; cat. no. 8242), pNF‐κB (1:1000, Cell Signaling Technology; cat. no. 3033), TNF‐α (1:1000, Abcam; cat. no. ab205587), and iNOS (1:1000, Abcam; cat. no. ab15323). The next day, the secondary antibody HRP‐labeled goat anti‐rabbit antibody (1:1000, Cell Signaling Technology; cat. no. 7074) was added to incubate the membranes at 4°C for 2 h. The immunoblots were visualized by an imaging densitometer (ImageQuant LAS 500, GE Healthcare Bio‐Sciences AB,). The band intensity was quantified through the software ImageJ. β‐actin was used as the control.

### 2.7 Double immunofluorescence labeling

After 24 h treatment, the rats were anesthetized and perfused with PBS and 4% paraformaldehyde subsequently. Then, the brains were harvested, fixed in 4% paraformaldehyde at 4°C overnight, dehydrated in graded sucrose, and cut into 10 μm‐thick slices. Blocked in 5% normal donkey serum (Abcam; cat. no. ab7475) at room temperature for 1 h, primary antibodies were then added to incubate the sections overnight at 4°C: CD31 (1:100, Abcam; cat. no. ab24590), Pink1 (1:100, Abcam; cat. no. ab23707), caspase‐1 (1:100, Abcam; cat. no. ab1872), caspase‐3 (1:100, Abcam; cat. no. ab179517), IL‐1β (1:100, Cell Signaling Technology; cat. no. 12703), IL‐1R1 (1:100, Santa Cruz Biotechnology; cat. no. sc‐689), Iba1 (1: 100, Abcam; cat. no. ab15690), TNF‐α (1:100, Abcam; cat. no. ab205587), and iNOS (1:100, Abcam; cat. no. ab15323). Washed with PBS and the second antibodies were added into the slices to incubate for 1 h at room temperature. The secondary antibodies include Alexa Fluor^®^ 488‐conjugated goat anti‐mouse IgG (1:200, Thermo Fisher Scientific; A‐28175), Alexa Fluor^®^ 555‐conjugated donkey anti‐rabbit IgG (1:200, Thermo Fisher Scientific; A‐31572). Then, sealed with fluorescent mounting medium (Sigma, St. Louis; cat. no. F6057), the slices were finally observed under the fluorescence microscope.

### 2.8 Enzyme‐linked Immunosorbent Assay (ELISA)

A commercial ELISA kit (Abcam; cat. no. ab100768) was used to quantify the expression of IL‐1β. After 24 h treatment, the rats were sacrificed under deep anesthesia. The hippocampus was collected on ice. All the procedures were carried out according to the manufactory's introduction.

### 2.9 Transmission electron microscope observation

Transmission electron microscope (TEM) observation was applied to detect the morphological changes of mitochondria. After 24 h treatment, the rats were sacrificed by decapitation under deep anesthesia. The hippocampus was removed and immersed in glutaraldehyde. The hippocampal tissues were cut into 1 mm^3^ and immersed in 1% osmium tetroxide for 3 h. Then, the tissue samples were washed three times in PBS, dehydrated with graded ethanol, and immersed in epoxy. Finally, the hippocampal tissues were cut into ultrathin sections, stained with uranyl acetate and lead citrate, and observed under the transmission electron microscope.

### 2.10 Statistical analysis

Statistical analysis was performed using SPSS version 19.0. All values were expressed as means ± standard deviations. Shapiro‐Wilk test was used to assess the data distribution. All data were normally distributed in the present study. Student's t‐test was used to analyze two‐group univariate‐factor measurement data. One‐way analysis of variance (ANOVA) was used to analyze the data of three‐ or four‐group univariate‐factor measurements. Repeated measures ANOVA was used to analyze the repeated measurement data. Following ANOVA, multiple comparisons were performed using Tukey's test. *p* < 0.05 was considered to indicate a statistically significant difference.

## 3 RESULTS

### 3.1 Fisetin improved cognitive impairment in CLP rats

**FIGURE 1:**
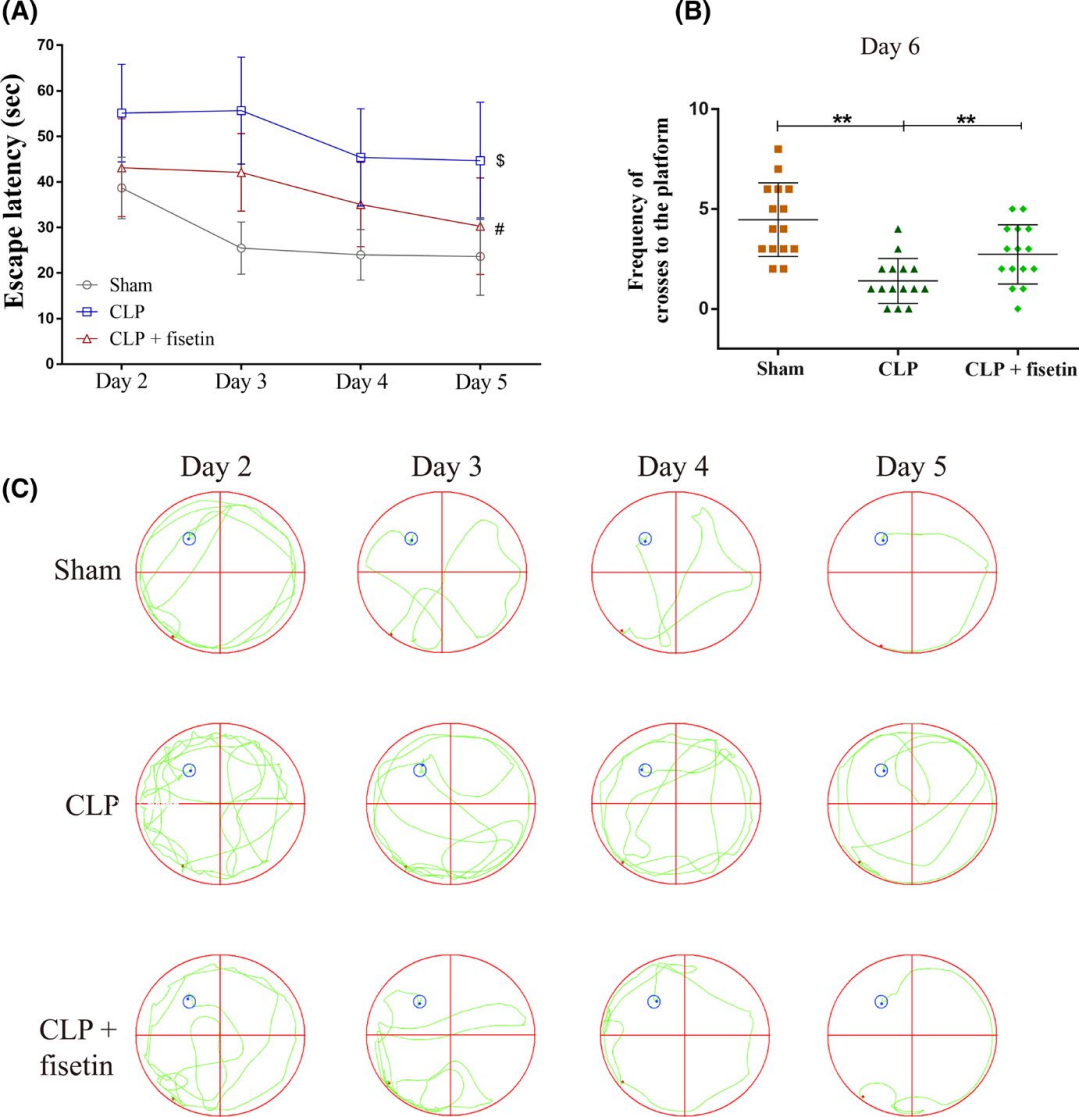
Fisetin improved cognitive impairment in CLP rats. (A) Escape latency of the sham, CLP, and CLP + fisetin rats decreased from day 2 to day 5. CLP treatment increased escape latency of sham rats, while fisetin intervention decreased escape latency of CLP rats. (B) The frequency of cross to the platform of the sham, CLP, and CLP + fisetin rats was recorded on day 6. CLP treatment decreased the frequency of sham rats, while fisetin intervention increased the frequency of CLP rats. (C) Escape passage of the sham, CLP, and CLP + fisetin rats from day 2 to day 5 was labeled. CLP treatment increased the escape passage of sham rats, while fisetin intervention decreased the escape passage of CLP rats. ^#^ *p* < 0.01 versus sham group, ^*^ *p* < 0.01 versus CLP group_,_^**^ *p* < 0.01

The escape latency of each group was longest on day 2 and reduced from day 3 to day 5. From day 2 to day 5, it was observed that the escape latency was increased in the CLP group compared to the sham group (^$^ *p* < 0.01) while the decreased one in the CLP + fisetin group compared to the CLP group (^#^ *p* < 0.01) (Figure 1A). The maximum frequency of crossing the platform on day 6 was observed in the sham group. The frequency was decreased in the CLP group compared to the sham group (^**^ *p* < 0.01) and less decreased in the CLP + fisetin group (^**^ *p* < 0.01) (Figure 1B). Similarly, the escape passage of each group was longest on day 2 and reduced from day 3 to day 5. From day 2 to day 5, the CLP group presented increased escape passage compared to the sham group, while the escape passage was shortened in the CLP + fisetin group compared to the CLP group (Figure 1C).

### 3.2 CLP triggers neuronal apoptosis without BBB impairment in 24 h

**FIGURE 2:**
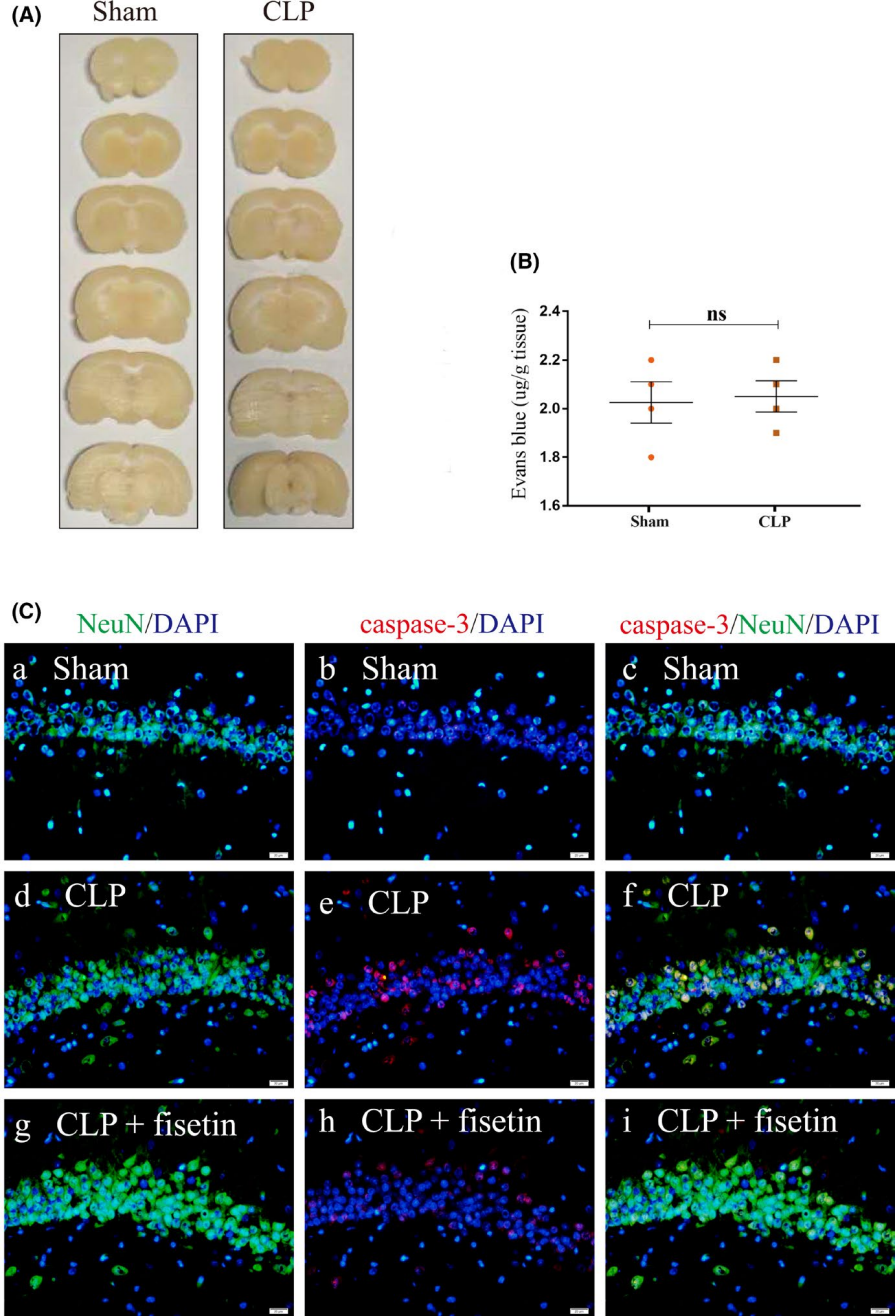
CLP triggers neuronal apoptosis without BBB impairment in 24 h. (A) The leakage of EB could not be observed in cerebral coronary sections. (B) There was no significant difference in the content of EB between the sham group and the CLP group. ns: no significant. (C) The immunofluorescence images show the expression of caspase‐3 (b, e, h, red), NeuN^+^ neurons (a, d, g, green), and the co‐localization of caspase‐3 and neurons (c, f, i). The fluorescence intensity of caspase‐3 in the CLP group was markedly elevated compared to the sham group. Conversely, the fluorescence intensity of caspase‐3 was markedly attenuated in the CLP + fisetin group compared to the CLP group. BBB, blood‐brain barrier; EB, Evan's blue. ^ns^ *p* > 0.05. Scale bars: 20 μm. ns: no significant

Little leakage of EB could be observed in the cerebral coronal section of the sham and CLP groups (Figure 2A). Besides, there was no significant difference in the content of EB between the sham and CLP groups (^ns^ *p* > 0.05) (Figure 2B). The immunofluorescence co‐localization technique was used to analyze neuronal apoptosis. The fluorescence intensity of caspase‐3 in the CLP group was markedly enhanced compared to the sham group. Conversely, the fluorescence intensity of caspase‐3 was markedly attenuated in the CLP + fisetin group compared to the CLP group (Figure 2C).

### 3.3 Fisetin upregulated the expression of Pink1 and Parkin in CMECs of CLP rats

**FIGURE 3:**
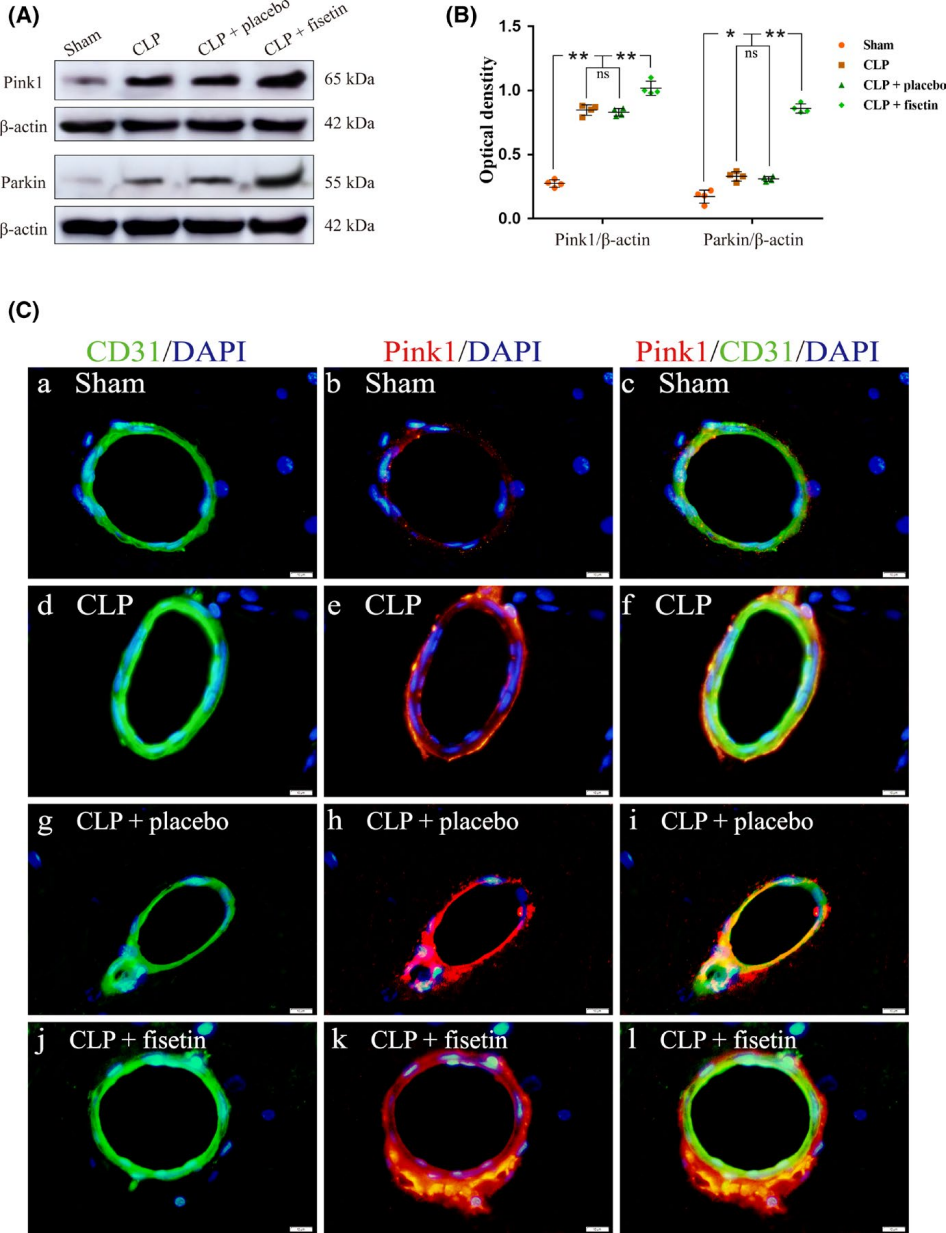
Fisetin upregulated the expression of Pink1 and Parkin in CMECs of CLP rats. (A) The immunoreactive bands of Pink1 (65 kDa), Parkin (55 kDa), and β‐actin (42 kDa). (B) The bar graph showed the increased expression of Pink1 and Parkin in the CLP group compared to the sham group, and the expression of Pink1 and Parkin was further upregulated in the CLP + fisetin group compared to the CLP group. There was no significant difference in the expression of Pink1 and Parkin between the CLP and CLP + placebo group. (C) The immunofluorescence images show the expression of Pink1 (b, e, h, k, red), CD31^+^ CMECs (a, d, g, j, green), and the co‐localization of Pink1 and CMECs (c, f, i, l). The fluorescence intensity of Pink1 in the CLP group was markedly elevated compared to the sham group. Besides, the fluorescence intensity of Pink1 was further elevated in the CLP + fisetin group, but not in the CLP + placebo group compared to the CLP group. ^*^ *p* < 0.05, ^**^ *p* < 0.01, ^ns^ *p* > 0.05. Scale bars: 10 μm. ns: no significant

The expression levels of Pink1 and Parkin in CMECs were detected by western blot (Figure 3A). The expression levels of Pink1 and Parkin were upregulated in the CLP group compared with those in the sham group (Pink1: ^**^ *p* < 0.01, Parkin: ^*^ *p* < 0.05). Furthermore, the expression levels of Pink1 and Parkin were significantly increased in the CLP + fisetin group (Pink1: ^**^ *p* < 0.01, Parkin: ^**^ *p* < 0.01), but not in the CLP + placebo group compared with those in the CLP group (^ns^ *p* > 0.05) (Figure 3B). The immunofluorescence co‐localization technique was used to analyze the expression of Pink1 in CMECs. The fluorescence intensity of Pink1 in the CLP group was markedly enhanced compared to the sham group. Furthermore, the fluorescence intensity of Pink1 was markedly enhanced in the CLP + fisetin group, but not in the CLP + placebo group compared to the CLP group (Figure 3C).

### 3.4 Fisetin promoted mitophagy through upregulating LC3‐II, downregulating p62, reducing ROS in CMECs of CLP rats

**FIGURE 4:**
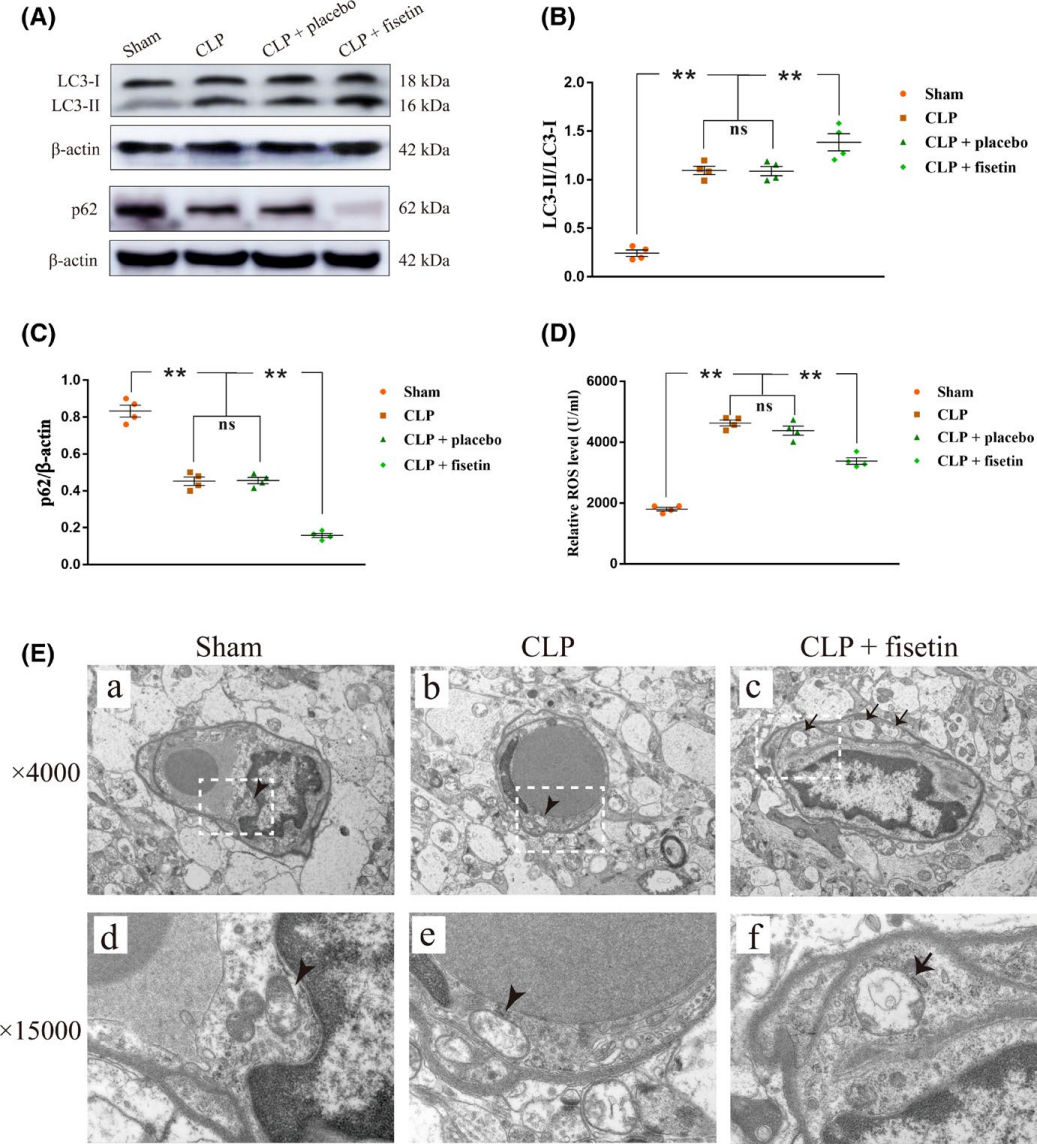
Fisetin promoted mitophagy through upregulating LC3‐II, downregulating p62, overproducing ROS in CMECs of CLP rats. (A) The immunoreactive bands of LC3‐I (18 kDa), LC3‐II (16 kDa), p62 (62 kDa), and β‐actin (42 kDa). (B) The bar graph showed increased expression of the ratio of LC3‐II to LC3‐I in the CLP group compared to the sham group, and the expression of Pink1 and Parkin was further upregulated in the CLP + fisetin group compared to the CLP group. There was no significant difference in expression of the ratio of LC3‐II to LC3‐I between the CLP and CLP + placebo groups. (C) The bar graph showed the decreased expression of p62 in the CLP group compared to the sham group, and the expression of p62 was further downregulated in the CLP + fisetin group compared to the CLP group. There was no significant difference in the expression of p62 between the CLP and CLP + placebo groups. (D) The bar graph showed the increased level of ROS in the CLP group compared to the sham group, but the level of ROS was significantly downregulated in the CLP + fisetin group compared to the CLP group. There was no significant difference in the level of ROS between the CLP and CLP + placebo groups. (E) TEM images of mitochondria in CMECs. Compared to the sham group (a, d), impaired mitochondria in the CLP group were swelling and vacuolar (b, e, arrowhead). Conversely, impaired mitochondria were rarely observed and autophagosomes were easily detected after treatment with fisetin (c, f, arrow) compared to the CLP group. ^**^ *p* < 0.01, ^ns^ *p* > 0.05. ns: no significant

The expression levels of LC3‐I, LC3‐II, and p62 in CMECs were detected by western blot (Figure 4A). The ratio of LC3‐II to LC3‐I was upregulated in the CLP group compared to the sham group (^**^ *p* < 0.01). They were significantly increased in the CLP + fisetin group (^**^ *p* < 0.01) (Figure 4B). The expression level of p62 was downregulated in the CLP group compared to those in the sham group (^**^ *p* < 0.01) while it was significantly decreased in the CLP + fisetin group (^**^ *p* < 0.01) (Figure 4C). The expression level of ROS in the CLP group was upregulated compared to the sham group (^**^ *p* < 0.01). Conversely, the expression level of ROS was significantly downregulated in the CLP + fisetin group (^**^ *p* < 0.01) (Figure 4D). The ratio of LC3‐II to LC3‐I, p62, and ROS showed no significance between the CLP + placebo group and the CLP group (^ns^ *p* > 0.05) (Figure 4B–D). Transmission electron microscopy was used to examine the benefits of fisetin on the mitochondrial structure. Impaired mitochondria (e, arrowhead) were swelling and vacuolar in the CLP group compared to the sham group. Conversely, impaired mitochondria were rarely observed and autophagosomes were easily detected after treatment with fisetin (f, arrow) compared to the CLP group (Figure 4E).

### 3.5 Fisetin‐blocked activation of NLRP3 inflammasome in CMECs of CLP rats

**FIGURE 5:**
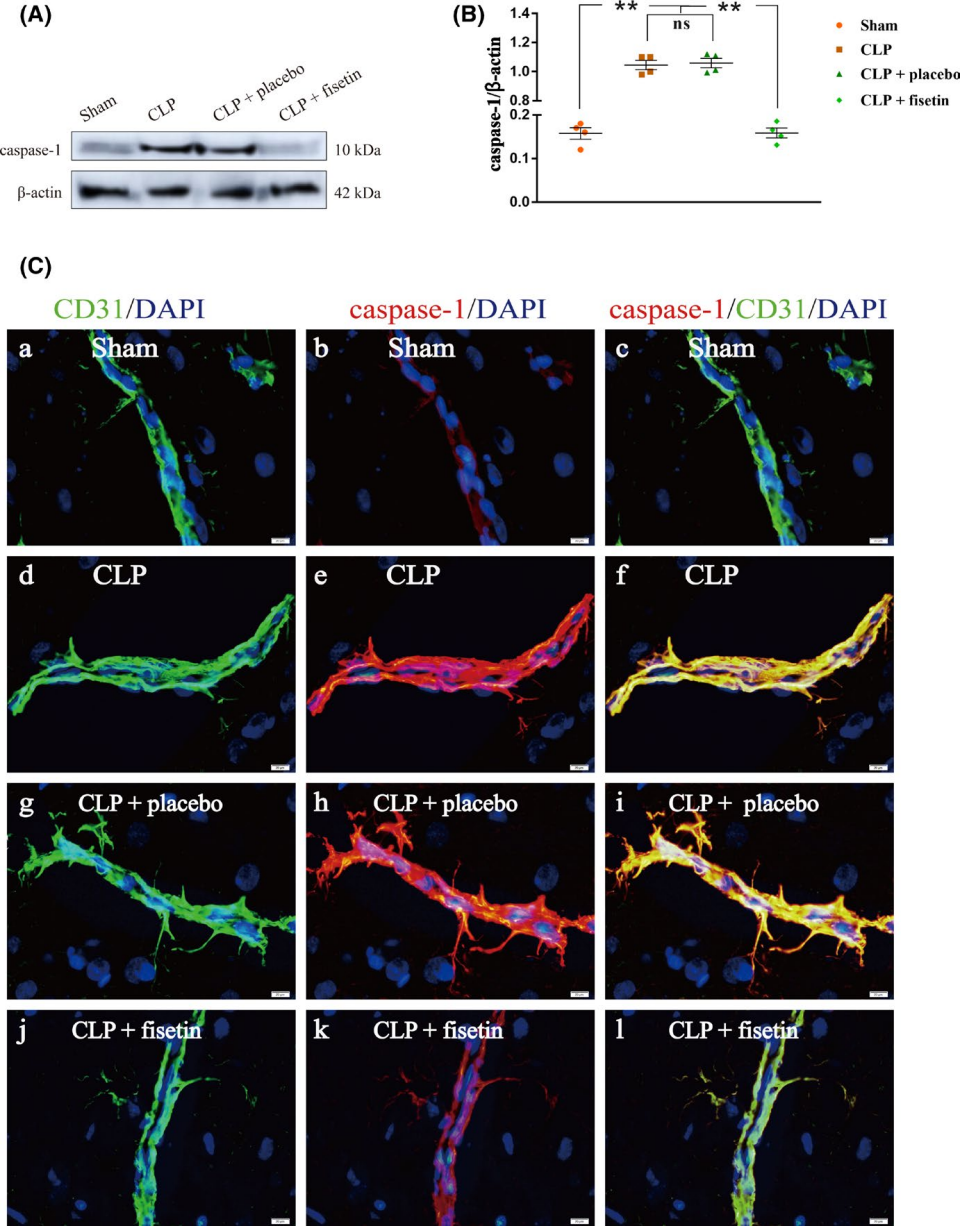
Fisetin‐blocked activation of NLRP3 inflammasome in CMECs of CLP rats. (A) The immunoreactive bands of caspase‐1 (10 kDa) and β‐actin (42 kDa). (B) The bar graph showed the upregulated expression of caspase‐1 in the CLP group compared to the sham group. Conversely, the expression levels of caspase‐1 were significantly downregulated in the CLP + fisetin group, but not in the CLP + placebo group compared with those in the CLP group. (C) The immunofluorescence images show the expression of caspase‐1 (b, e, h, k, red), CD31^+^ CMECs (a, d, g, j, green), and the co‐localization of caspase‐1 and CMECs (c, f, i, l). The fluorescence intensity of caspase‐1 in the CLP group was markedly elevated compared to the sham group. Conversely, the fluorescence intensity of caspase‐1 was markedly attenuated in the CLP + fisetin group, but not in the CLP + placebo group compared to the CLP group. ^**^ *p* < 0.01, ^ns^ *p* > 0.05. Scale bars: 20 μm. ns: no significant

The expression level of caspase‐1 in CMECs was detected by western blot (Figure 5A). The expression level of caspase‐1 in the CLP group was upregulated compared with those in the sham group (^**^ *p* < 0.01). Conversely, the expression level of caspase‐1 was significantly downregulated in the CLP + fisetin group (^**^ *p* < 0.01), but not in the CLP + placebo group (^ns^ *p* > 0.05) compared with those in the CLP group (Figure 5B). The immunofluorescence co‐localization technique was used to analyze the expression of caspase‐1 in CMECs. The fluorescence intensity of caspase‐1 in the CLP group was markedly enhanced compared to the sham group. Conversely, the fluorescence intensity of caspase‐1 was markedly attenuated in the CLP + fisetin group, but not in the CLP + placebo group compared to the CLP group (Figure 5C).

### 3.6 Fisetin downregulated Il‐1β in CMECs of CLP rats

**FIGURE 6:**
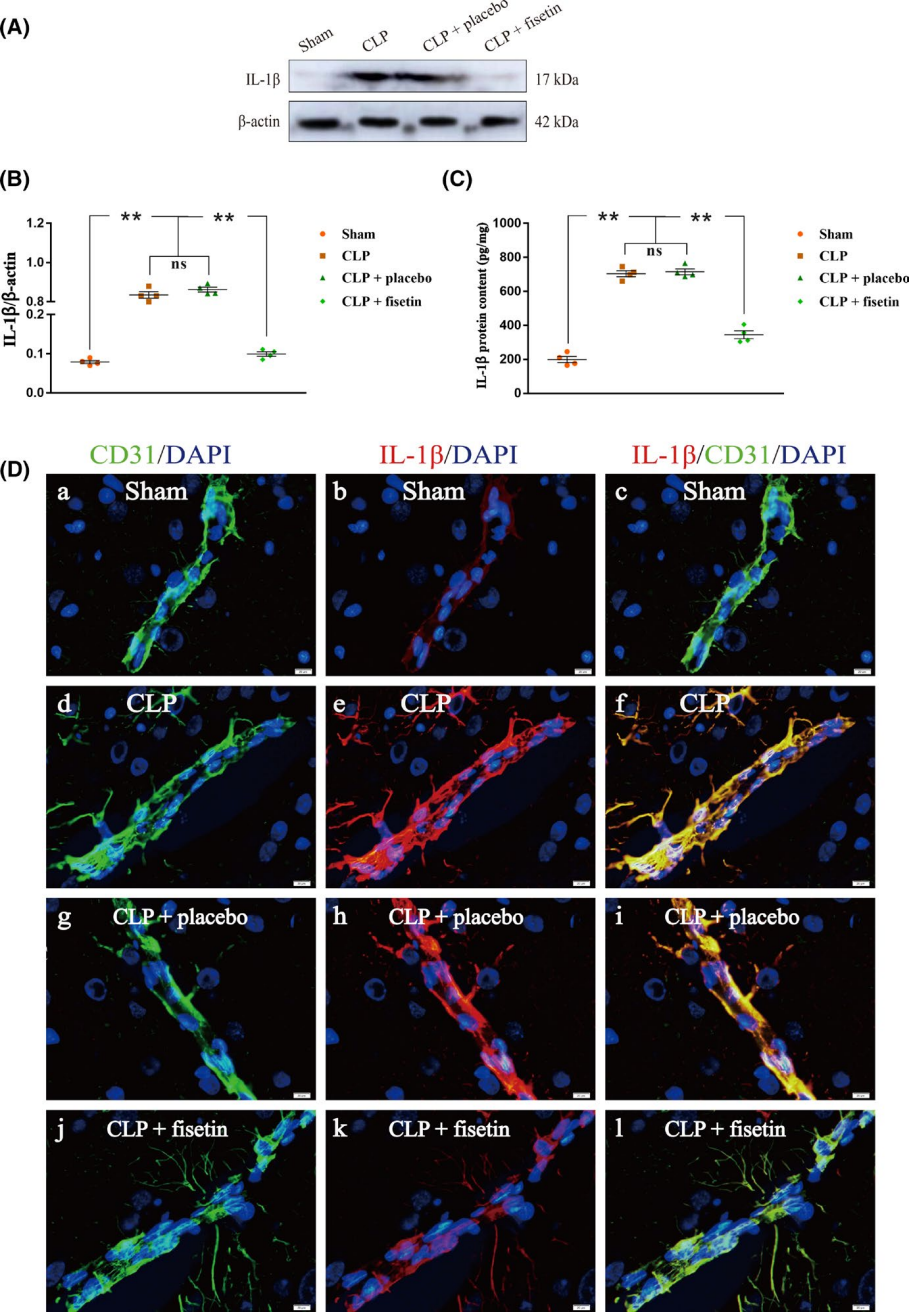
Fisetin downregulated IL‐1β in CMECs of CLP rats. (A) The immunoreactive bands of IL‐1β (17 kDa) and β‐actin (42 kDa). (B, C) The bar graph showed the upregulated expression of IL‐1β in the CLP group compared to the sham group. Conversely, the expression levels of IL‐1β were significantly downregulated in the CLP + fisetin group, but not in the CLP + placebo group compared with those in the CLP group. (D) The immunofluorescence images show the expression of IL‐1β (b, e, h, k, red), CD31^+^ CMECs (a, d, g, j, green), and the co‐localization of IL‐1β and CMECs (c, f, i, l). The fluorescence intensity of IL‐1β in the CLP group was markedly elevated compared to the sham group. Conversely, the fluorescence intensity of IL‐1β was markedly attenuated in the CLP + fisetin group, but not in the CLP + placebo group compared to the CLP group. ^**^ *p* < 0.01, *^ns^p* > 0.05. Scale bars: 20 μm. ns: no significant

The expression level of IL‐1β in CMECs was detected by western blot and ELISA, respectively (Figure 6A,B,C). The expression levels of IL‐1β in the CLP group were upregulated compared with those in the sham group (^**^ *p* < 0.01). Conversely, the expression levels of IL‐1β were significantly downregulated in the CLP + fisetin group (^**^ *p* < 0.01), but not in the CLP + placebo group compared with those in the CLP group (^ns^ *p* > 0.05) (Figure 6A,B). Results of the concentration of IL‐1β detected by an Elisa kit were in line with that by western blot (Figure 6C). The immunofluorescence co‐localization technique was used to analyze the expression of IL‐1β in CMECs. The fluorescence intensity of IL‐1β in the CLP group was markedly enhanced compared to the sham group. Conversely, the fluorescence intensity of IL‐1β was markedly attenuated in the CLP + fisetin group, but not in the CLP + placebo group compared to the CLP group (Figure 6D).

### 3.7 Fisetin downregulated IL‐1R1 and NF‐κB phosphorylation in microglial cells of CLP rats

The expression levels of IL‐1R1, NF‐κB, and pNF‐κB in microglial cells were detected by western blot (Figure S1A). The expression level of IL‐1R1 in the CLP group was upregulated compared with those in the sham group (^**^ *p* < 0.01). Conversely, the expression level of IL‐1R1 was significantly downregulated in the CLP + fisetin group (^**^ *p* < 0.01), but not in the CLP + placebo group (^ns^ *p* > 0.05) compared with those in the CLP group (Figure S1B). The expression level of NF‐κB presented no significant difference in the sham, CLP, CLP + placebo, and CLP + fisetin group (^ns^ *p* > 0.05) (Figure S1C). The expression level of pNF‐κB in the CLP group was upregulated compared with those in the sham group (^**^ *p* < 0.01). Conversely, the expression level of pNF‐κB was significantly downregulated in the CLP + fisetin group (^**^ *p* < 0.01), but not in the CLP + placebo group compared with those in the CLP group (*^ns^p* > 0.05) (Figure S1D). The immunofluorescence co‐localization technique was used to analyze the expression of IL‐1R1 in microglial cells. The fluorescence intensity of IL‐1R1 in the CLP group was markedly enhanced compared to the sham group. Conversely, the fluorescence intensity of IL‐1R1 was markedly attenuated in the CLP + fisetin group, but not in the CLP + placebo group compared to the CLP group (Figure S1E).

### 3.8 Fisetin downregulated TNF‐α and iNOS in microglial cells of CLP rats

The expression levels of TNF‐α and iNOS in microglial cells were detected by western blot (Figure S2A and S3A). The expression levels of TNF‐α and iNOS in the CLP group were upregulated compared with those in the sham group (^**^ *p* < 0.01). Conversely, the expression levels of TNF‐α and iNOS were significantly downregulated in the CLP + fisetin group (^**^ *p* < 0.01), but not in the CLP + placebo group compared with those in the CLP group (^ns^ *p* > 0.05) (Figure S2B and S3B). The immunofluorescence co‐localization technique was used to analyze the expression of TNF‐α and iNOS in microglial cells. The fluorescence intensity of TNF‐α and iNOS in the CLP group was markedly enhanced compared to the sham group. Conversely, the fluorescence intensity of TNF‐α and iNOS was markedly attenuated in the CLP + fisetin group, but not in the CLP + placebo group compared to the CLP group (Figure S2C and S3C).

## 4 DISCUSSION

Fisetin is recommended to be active therapeutic in many disorders, including neurological diseases, while its role in SAE is still unclear.^16^, ^17^, ^18^, ^19^ In this study, we confirmed the neuroprotective effects of fisetin on cognitive deficits caused by SAE. Furthermore, we explored the potential mechanism of fisetin on SAE, which might be relevant to increased mitophagy, scavenged ROS, inactivated NLRP3 inflammasome in CMECs, along with decreased caspase‐1 levels, and reduced secretion of IL‐1β into CNS.

Fisetin is documented to be beneficial for many diseases, such as cancers, cardiovascular disease, neurodegenerative disorders, and so on.^20^, ^21^ However, the bioactive potential of fisetin in sepsis and SAE has not been completely elaborated. Sepsis is a common but lethal cause for multiple organ dysfunction, and SAE is the pathological change when septic damages spread to the brain, characterized by diffused brain dysfunction.^22^ Based on recent studies, many brain areas, including the cortex, thalamus, hippocampus, striatum, basal ganglia, and corpus callosum could be affected by CLP.^23^, ^24^, ^25^ Of note, the hippocampus region has a close association with cognitive impairment,^26^ so the hippocampus tissue was selected for our research. As demonstrated in this study, septic rats showed significant cognitive deficits, including prolonged escape latency, increased escape passage, reduced frequency of cross to the platform, and enlarged neuronal apoptosis. In order to investigate the effect of fisetin on SAE, therefore, fisetin was used to treat the septic rats. Notably, fisetin presented neuroprotective properties since it attenuated the cognitive impairment in rats with SAE.

Although the pathological mechanism of SAE is versatile and complex, to date, neuroinflammation is suggested to be responsible for cognitive impairment in patients with SAE.^27^, ^28^, ^29^ Microglial cells are the resident macrophages in the CNS. Activation of microglial cells along with the aberrant release of proinflammatory factors leads to the exaggeration of local inflammatory response and finally precipitates neuronal injuries and cognitive dysfunction.^30^, ^31^ In this study, rats with CLP exhibited cognitive deficits 24 h after surgery, as well as severe inflammation in the CNS, manifested as increased expression of IL‐1R1, pNF‐κB, TNF‐α, and iNOS in microglial cells. However, there was no increase in the BBB permeability. It remains unclear how peripheral inflammation can be transmitted to the CNS when BBB is intact.

In the present study, NLRP3 inflammasome was proved to be activated in CMECs of septic rats, as evidenced by an elevation of caspase‐1 expression and IL‐1β secretion into CNS. The results indicated that septic injury induced the activation of NLRP3 inflammasome in CMECs and the increased release of IL‐1β. Under the pathological condition, NLRP3 inflammasome is activated through two steps: priming and assembly.^32^ Take gram‐negative sepsis as an example, lipopolysaccharide (LPS) promotes translocation of NF‐κB into the nucleus, and then NLRP3 and pro‐IL‐1β were activated at the transcriptional level. Then, it follows the activation of NLRP3 inflammasome assembly, which could be induced by a myriad of events, such as potassium efflux, ROS generation, lysosomal leakage, and so on. Ultimately, activated NLRP3 inflammasome aggravates inflammatory response via promoting the secretion of caspase‐1 and IL‐1β.^33^, ^34^, ^35^ In our study, CLP rats with pretreatment of fisetin blocked activation of NLRP3 inflammasome characterized by decreased expression of caspase‐1, which then suppressed the mature and secretion of IL‐1β in CMECs.

According to our results, little damage of the BBB could be observed after 24 h in rats with CLP surgery, which indicated that peripheral inflammatory cytokines such as IL‐1β were located and did not gain access to the CNS because the BBB was found to be functionally intact. Meanwhile, upregulated expression of IL‐1R1 was detected in microglial cells of CLP rats, which suggested the activation of IL‐1R1 and its upstream factor IL‐1β in the CNS. Taken together with the activation of NLRP3 inflammasome in CMECs of CLP rats, we hypothesized that IL‐1β in the microglia cells might derive from CMECs. To support our hypothesis, the expression of IL‐1β in CMECs was detected by western blot, ELISA, and immunofluorescence co‐localization technique. In line with upregulated IL‐1β in CMECs, microglial cells were activated through IL‐1R1/pNF‐κB pathway and resulted in neuroinflammatory response and cognitive impairment. Thereafter, decreased expression of IL‐1β in CMECs restricted its combination with IL‐1R1 in microglial cells, and thus inhibiting the transmitting of peripheral inflammation into the CNS.

Furthermore, our results also found the increased expression of Pink1 and Parkin as well as elevated activation of mitophagy in septic rats with fisetin administration, manifested by the upregulated ratio of LC3‐II/LC3‐I, decreased p62, increased ROS, and autophagosomes in CMECs. Under the physical status, mitophagy is fundamental for the elimination of damaged mitochondria and hemostasis of neurons which is highly dependent on aerobic metabolism.^36^ However, septic stimuli could promote this protective process mitophagy; then, the accumulation of ROS emanated from dysfunctional mitochondria give rise to activation of NLRP3 inflammasome and systemic inflammation, which was illustrated in our results of CLP rats. The interaction of mitophagy and NLRP3 inflammasome may be a conceivable therapeutic strategy in the treatment of sepsis.

We found that fisetin induced mitophagy, which promoted clearance of sepsis‐mediated damaged mitochondria and scavenging of ROS, furthermore, blocked NLRP3 inflammasome activation of CMECs and inhibited the release of IL‐1β into CNS. Meanwhile, the expression levels of IL‐1R1, pNF‐κB, TNF‐α, and iNOS were downregulated in microglial cells of the CNS. It is suggested that fisetin interacted directly with CMECs (the main component of BBB) to reduce the secretion of inflammatory factors (IL‐1β) into the CNS after sepsis, thereby attenuating central inflammation and cognitive impairment. This study provides additional insights of fisetin into the treatment of cognitive impairment induced by SAE.

The limitations of this study are as follows. It has been reported that oral administration of Fisetin could cross the BBB, reach the hippocampus, and improve memory impairment.^37^ However, in the present study, we did not test whether fisetin could pass the BBB in rats of the sham group and CLP group. Therefore, the possibility that Fisetin can act directly on activated microglia cannot be excluded. The lack of fisetin in microglia activation being investigated in our study is a limitation and also a direction for future research.

## 5 CONCLUSION

In conclusion, the present results suggest that fisetin‐blocked NLRP3 inflammasome activation via promoting mitophagy in CMECs may suppress the secretion of IL‐1β into the CNS, reduce neuroinflammation, and contribute to the amelioration of cognitive impairment. Specifically, fisetin promoted mitophagy via Pink1/Parkin signal in CMECs of rats with SAE, which contributed to increased elimination of ROS. Thereafter, activation of NLRP3 inflammasome was blocked and secretion of IL‐1β into the CNS in CMECs was inhibited, along with inactivation of microglial cells manifested as decreased levels of IL‐1R1, pNF‐κB, TNF‐α, and iNOS. Ultimately, fisetin administration managed to attenuate cognitive dysfunction induced by SAE through alleviating central neuroinflammation.

## CONFLICT OF INTEREST

The authors declare that the research was conducted in the absence of any commercial or financial relationships that could be construed as a potential conflict of interest.

## AUTHORS’ CONTRIBUTIONS

All authors have made significant contributions to this study. HGD was responsible for the conception proposal and manuscript review. YL, SLC, XSL, and HGD designed and conducted experiments. YW and SYZ took part in data collection and analysis. YL and WHZ contributed to drafting and revising this article. ESL, HKZ, and HGD gave financial support. All authors agree to submit and publish this article.

## Supporting information

Fig S1

Fig S2

Fig S3

Supplementary Material

Supplementary Material

## Data Availability

The authors confirm that the data supporting the findings of this study are available within this article and from the corresponding author upon reasonable request.
