# Supplementary figures and images for: Fisetin ameliorates cognitive impairment by activating mitophagy and suppressing neuroinflammation in rats with sepsis‐associated encephalopathy
Source: CNS Neurosci Ther. 2021 Nov 27;28(2):247–58. doi: 10.1111/cns.13765 (PMC8739041; doi:10.1111/cns.13765)

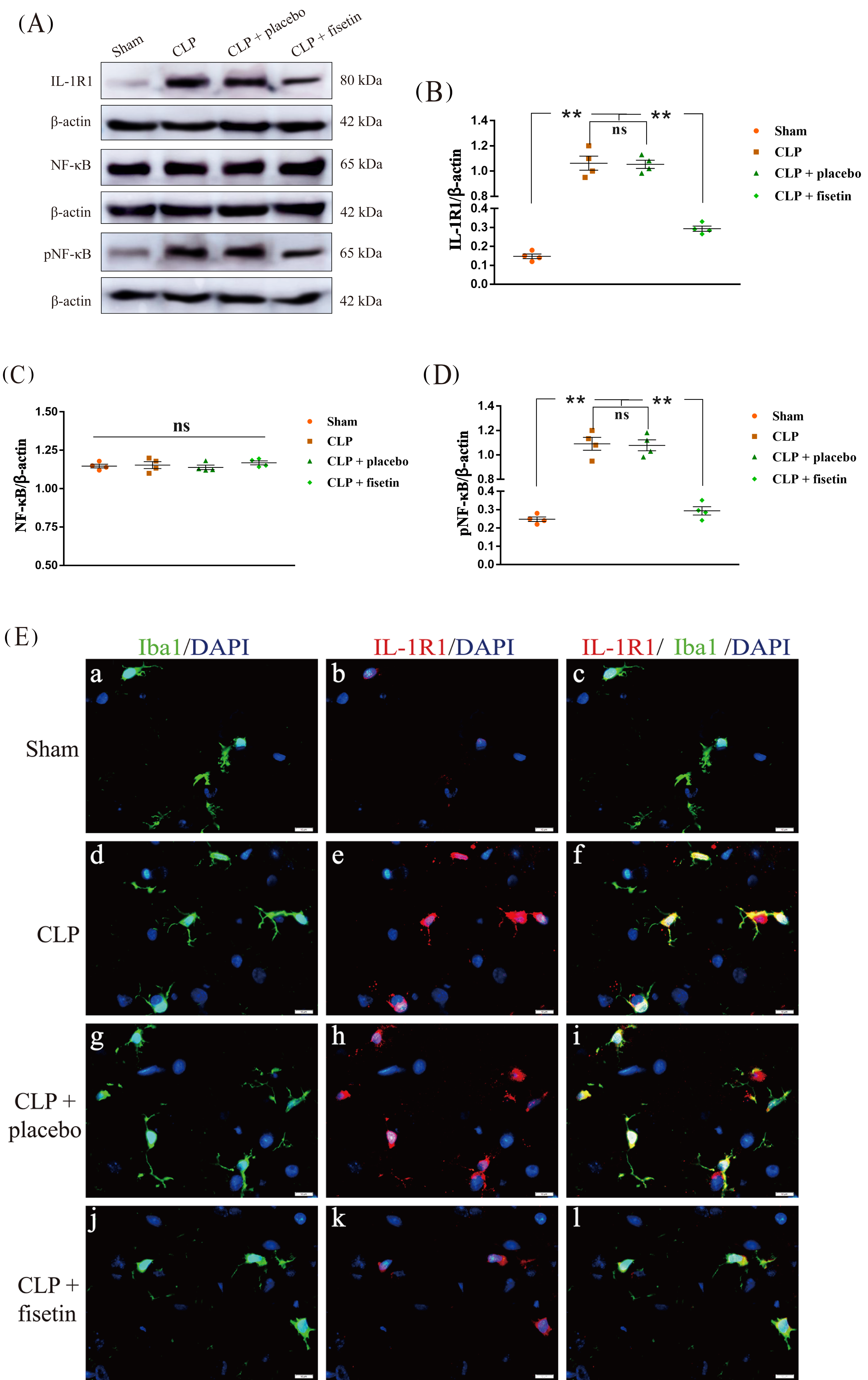

Supplement: Supplementary file 1 — Fig S1 [file CNS-28-247-s002.tif]

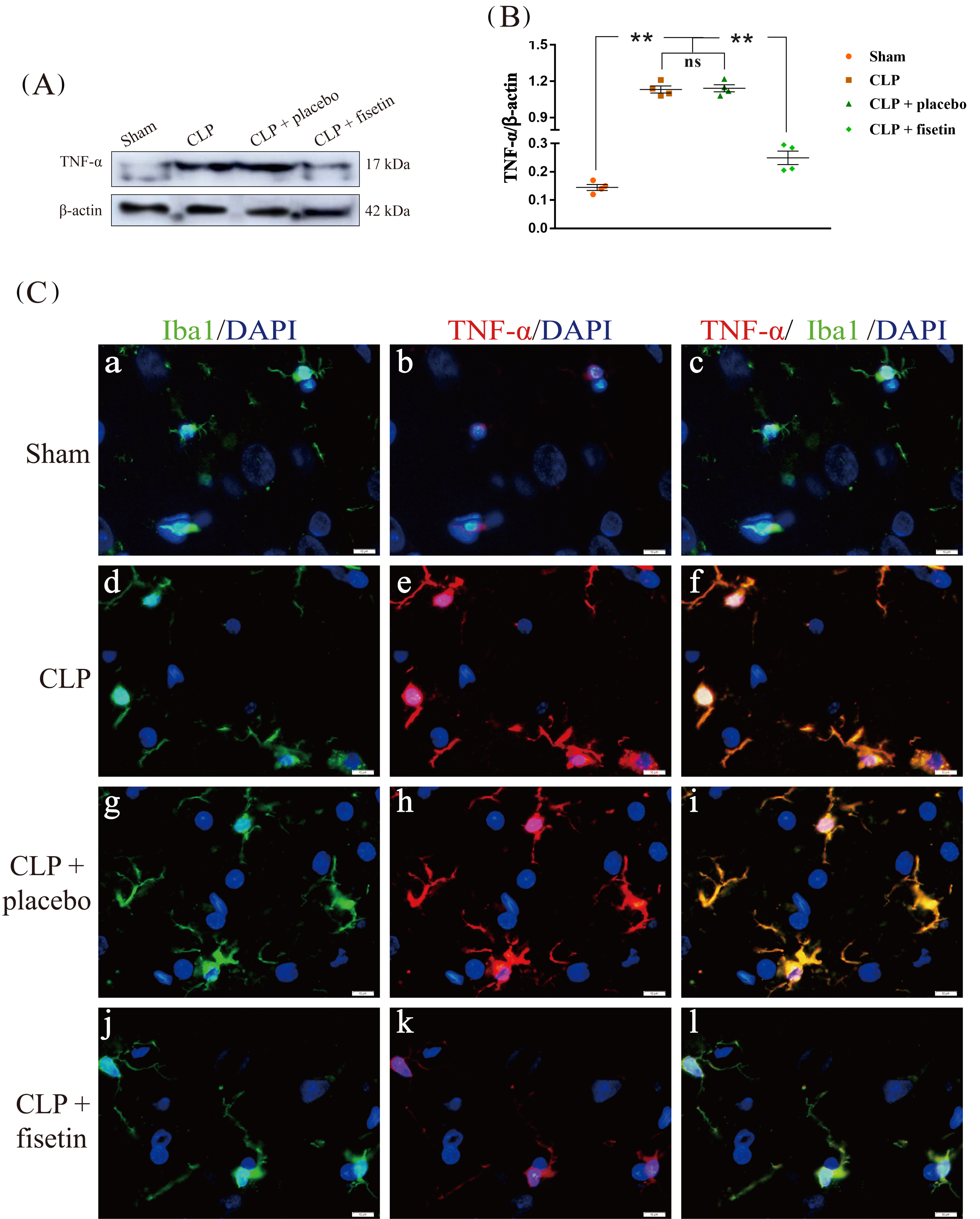

Supplement: Supplementary file 2 — Fig S2 [file CNS-28-247-s003.tif]

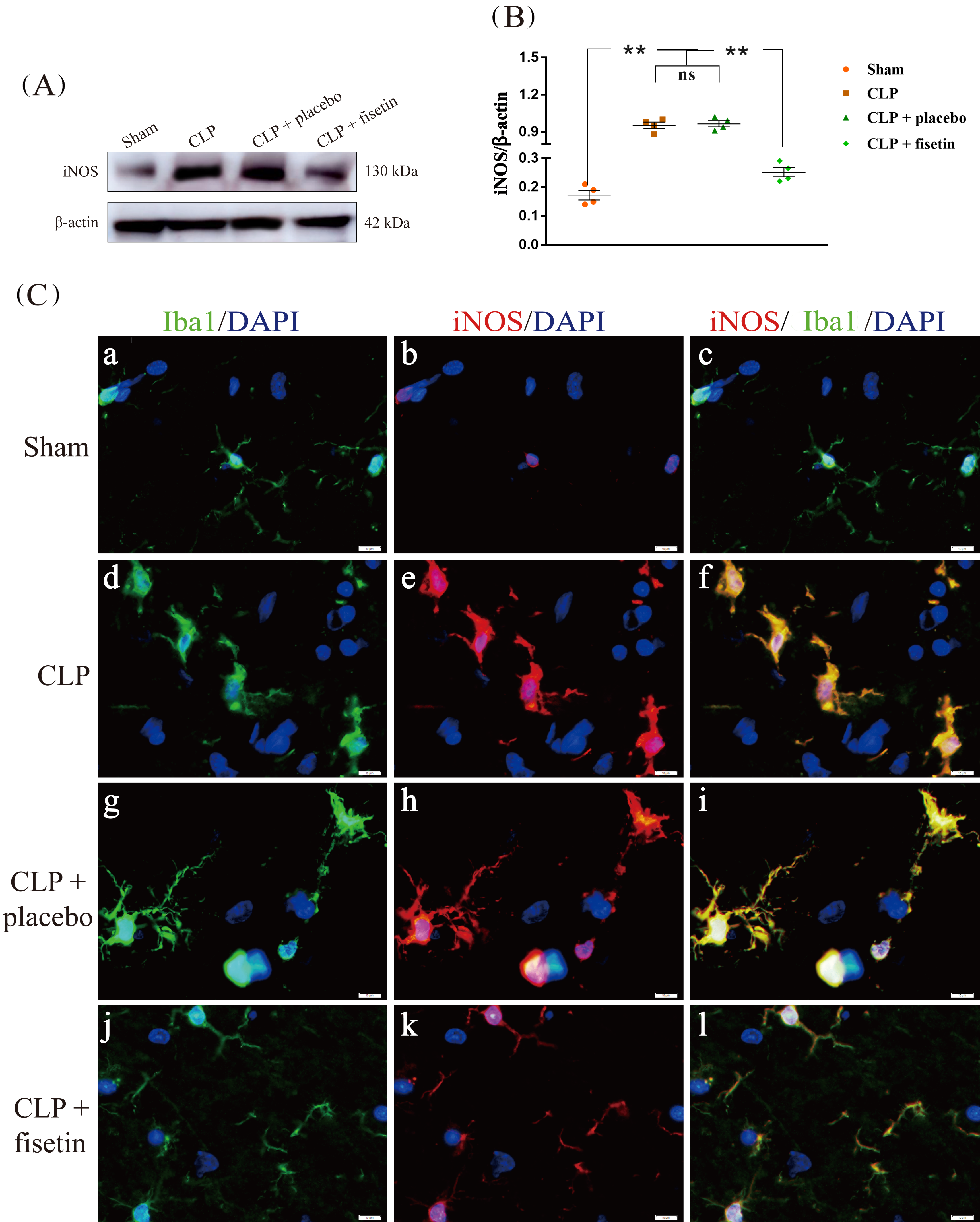

Supplement: Supplementary file 3 — Fig S3 [file CNS-28-247-s004.tif]
