# Supplementary material for: Fisetin ameliorates cognitive impairment by activating mitophagy and suppressing neuroinflammation in rats with sepsis‐associated encephalopathy
Source: CNS Neurosci Ther. 2021 Nov 27;28(2):247–58. doi: 10.1111/cns.13765 (PMC8739041; doi:10.1111/cns.13765)
Supplement: Supplementary file 4 — Supplementary Material [file CNS-28-247-s005.pdf]

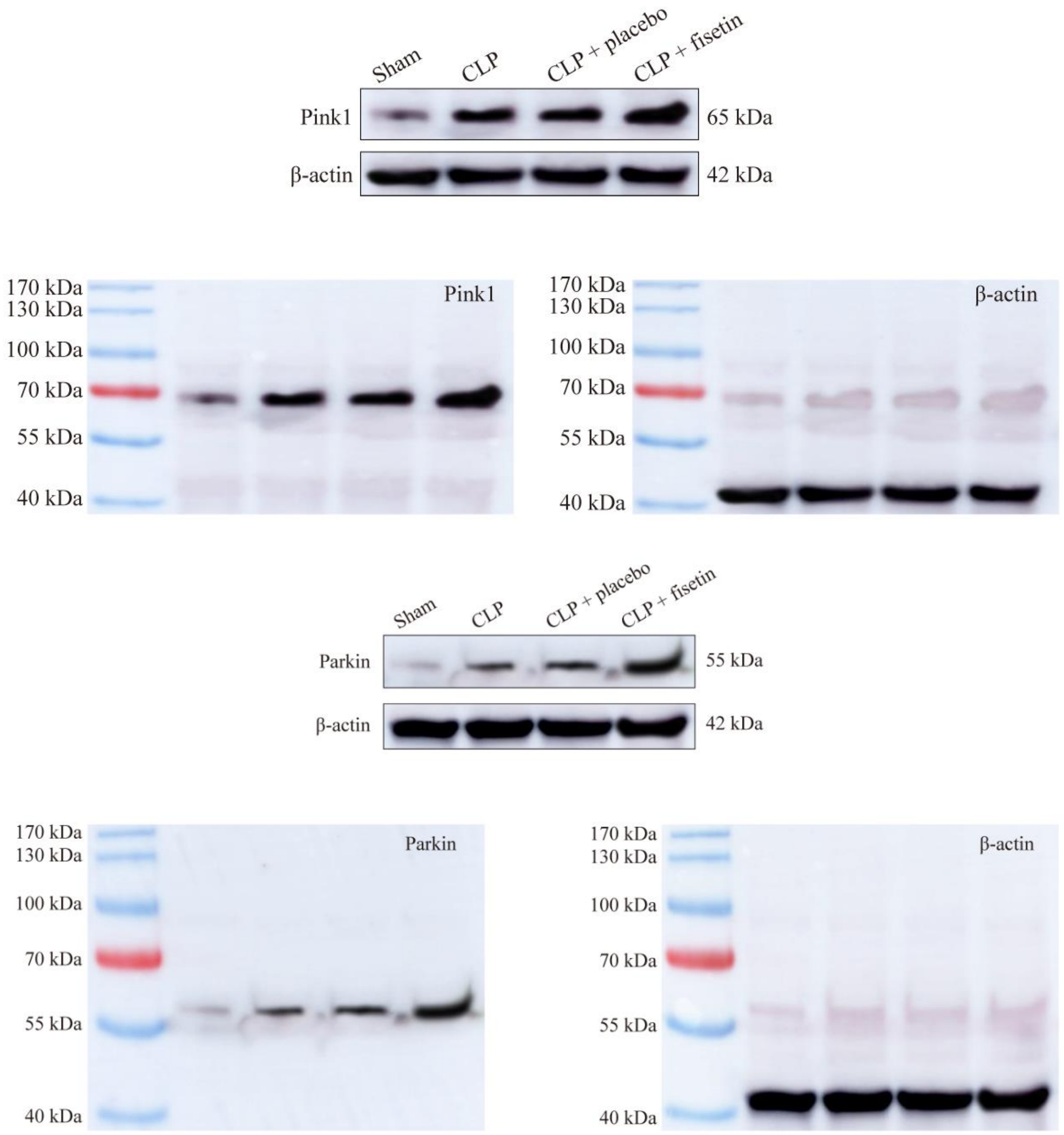

Full unedited blots for Figure 3.

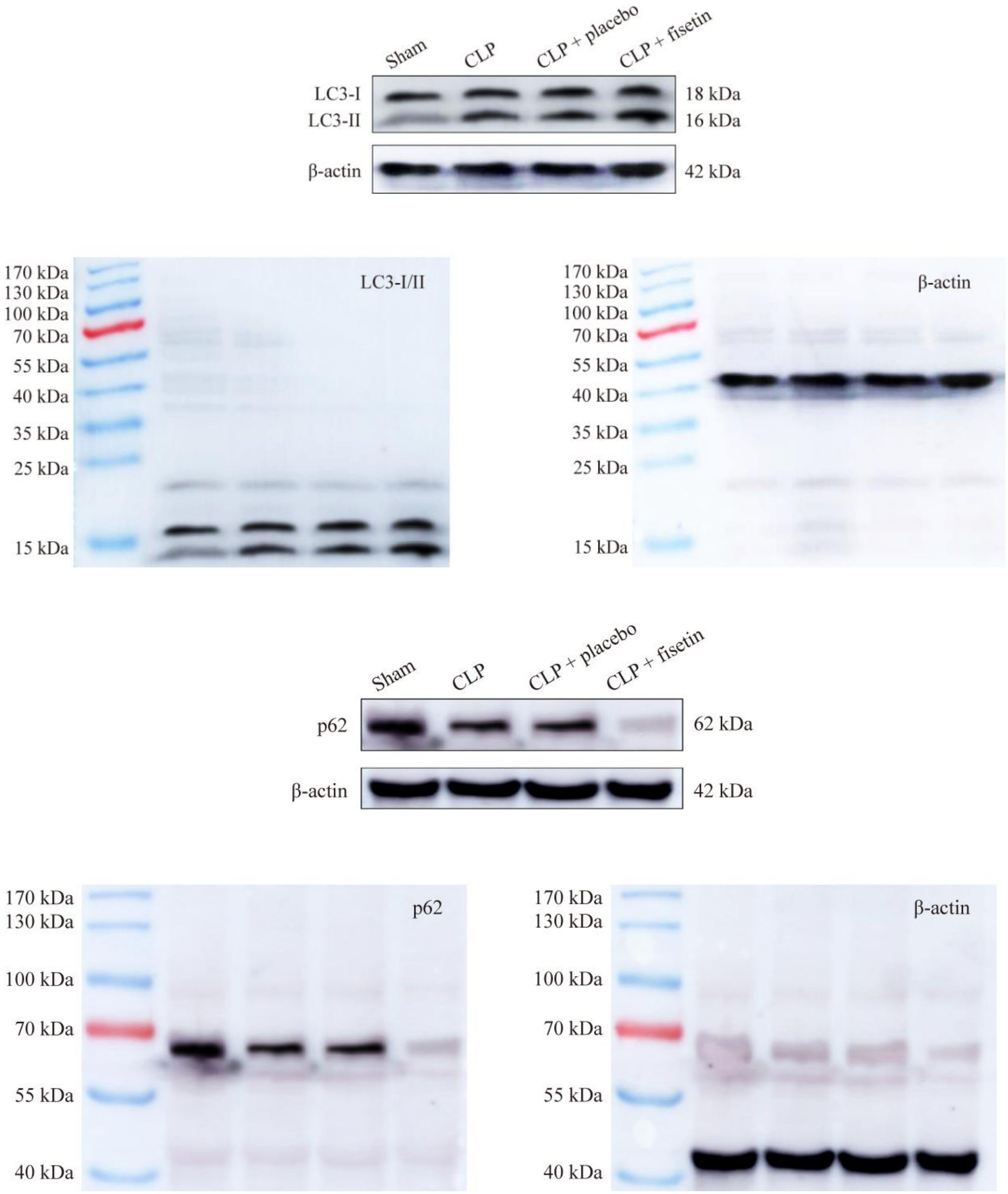

Full unedited blots for Figure 4.

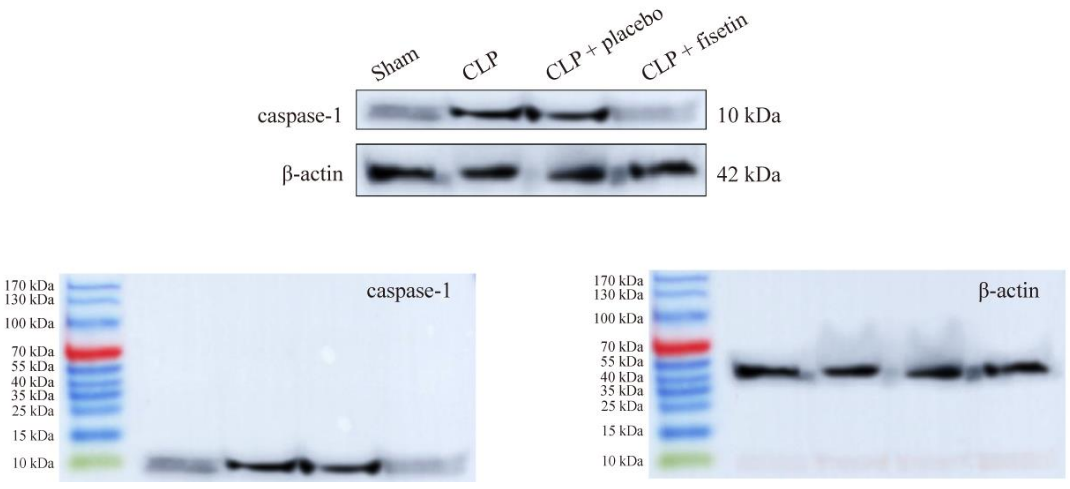

Full unedited blots for Figure 5.

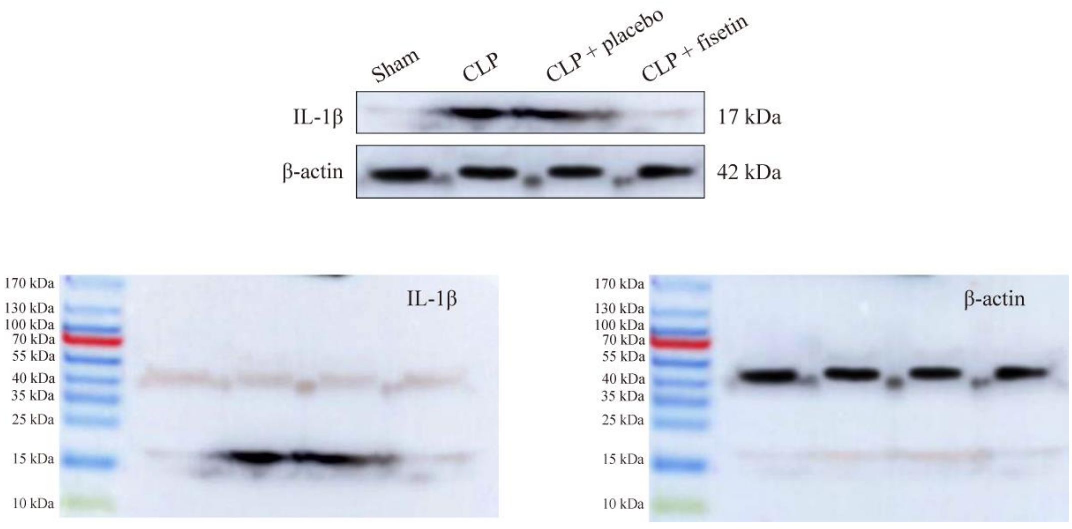

Full unedited blots for Figure 6.

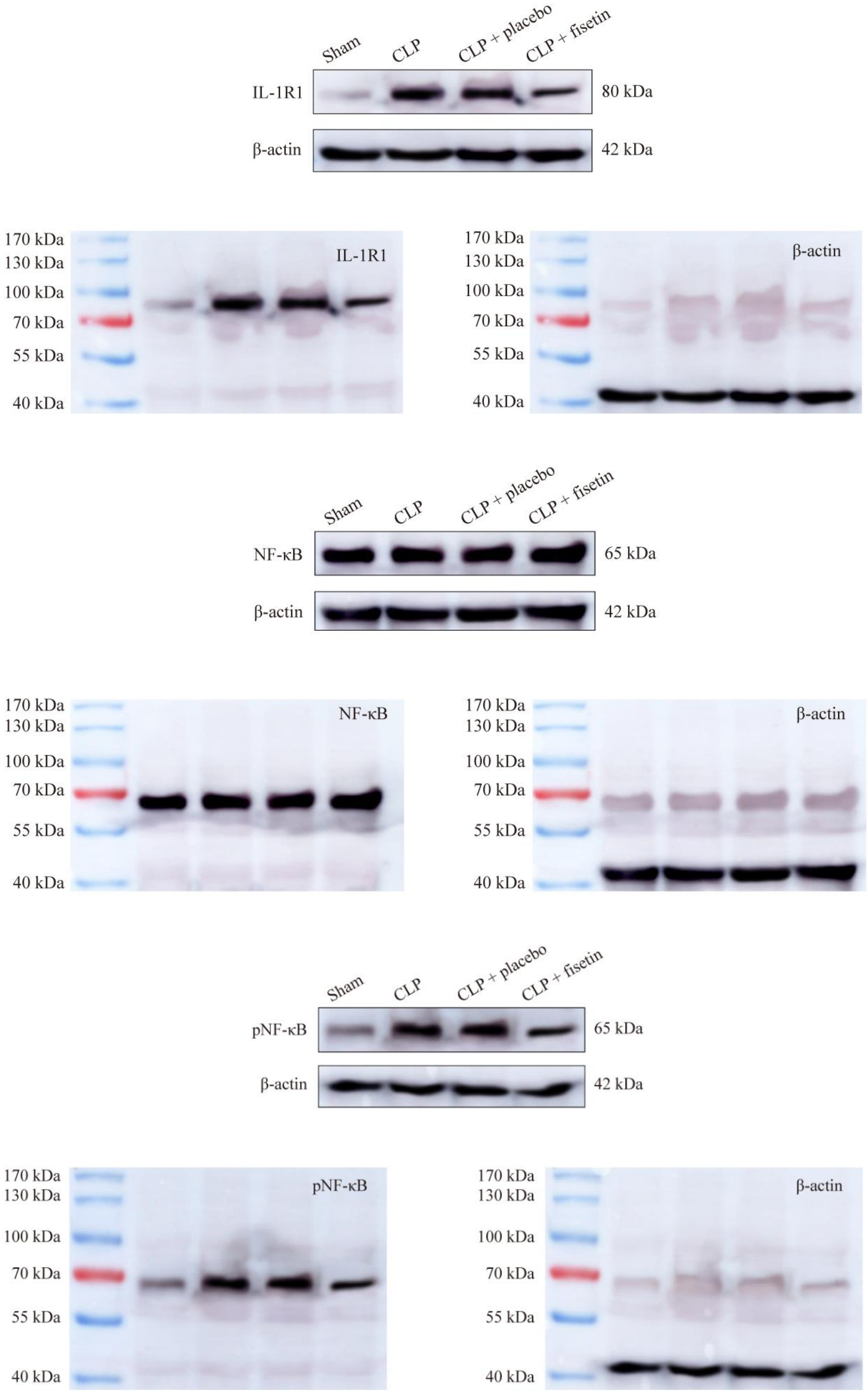

Full unedited blots for Figure S1

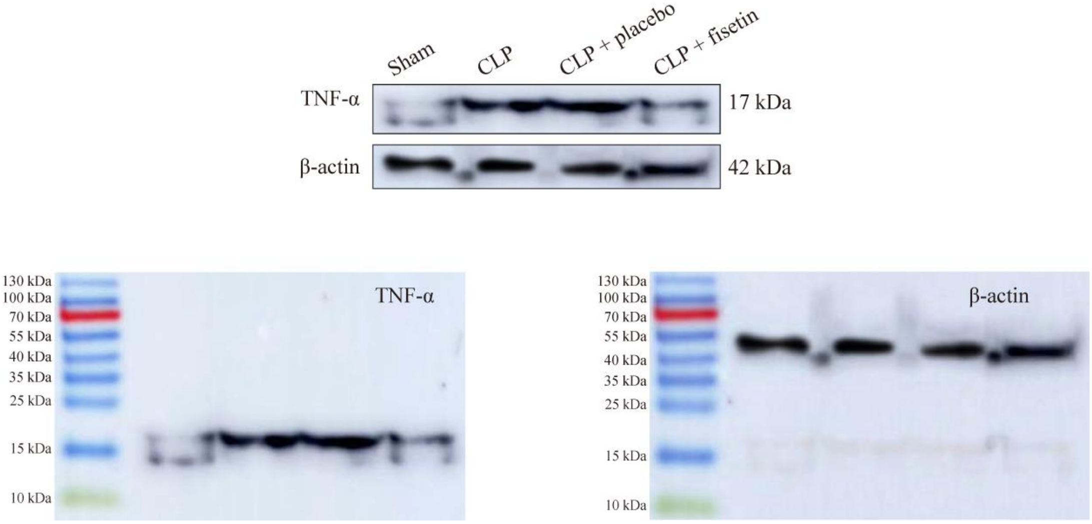

Full unedited blots for Figure S2.

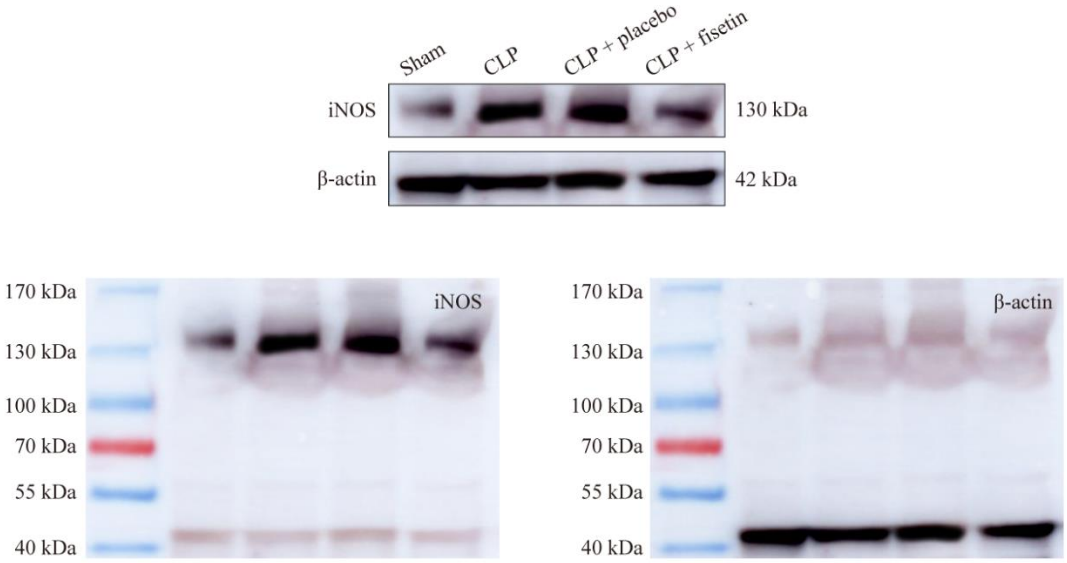

Full unedited blots for Figure S3.
