# Supplementary material for: Fisetin ameliorates cognitive impairment by activating mitophagy and suppressing neuroinflammation in rats with sepsis‐associated encephalopathy
Source: CNS Neurosci Ther. 2021 Nov 27;28(2):247–58. doi: 10.1111/cns.13765 (PMC8739041; doi:10.1111/cns.13765)
Supplement: Supplementary file 5 — Supplementary Material [file CNS-28-247-s001.docx]

**FIGURE S1** Fisetin downregulated IL-1R1 and NF-κB phosphorylation in microglial cells of CLP rats. (A) The immunoreactive bands of IL-1R1 (80 kDa), NF-κB (65 kDa), pNF-κB (65 kDa), and β-actin (42 kDa). (B) The bar graph showed the upregulated expression of IL-1R1 in the CLP group compared to the sham group. Conversely, the expression levels of IL-1R1 were significantly downregulated in the CLP+fisetin group, but not in the CLP+placebo group compared with those in the CLP group. (C) The bar graph showed the expression of NF-κB in the sham, CLP, CLP+placebo, and CLP+fisetin groups. There was no significant difference in the expression of NF-κB in each group. (D) The bar graph showed the upregulated expression of pNF-κB in the CLP group compared to the sham group. Conversely, the expression levels of pNF-κB were significantly downregulated in the CLP+fisetin group, but not in the CLP+placebo group compared with those in the CLP group. (E) The immunofluorescence images show the expression of IL-1R1 (b, e, h, k, red), Iba1^+^ microglial cells (a, d, g, j, green), and the co-localization of IL-1R1 and microglial cells (c, f, i, l). The fluorescence intensity of IL-1R1 in the CLP group was markedly elevated compared to the sham group. Conversely, the fluorescence intensity of IL-1R1 was markedly attenuated in the CLP+fisetin group, but not in the CLP+placebo group compared to the CLP group. ^**^P<0.01, ^ns^P>0.05. Scale bars: 10 μm. ns: no significant.

**FIGURE S2** Fisetin downregulated TNF-α in microglial cells of CLP rats. (A) The immunoreactive bands of TNF-α (17 kDa) and β-actin (42 kDa). (B) The bar graph showed the upregulated expression of TNF-α in the CLP group compared to the sham group. Conversely, the expression levels of TNF-α were significantly downregulated in the CLP+fisetin group, but not in the CLP+placebo group compared with those in the CLP group. (C) The immunofluorescence images show the expression of TNF-α (b, e, h, k, red), Iba1^+^ microglial cells (A, D, G, J, green), and the co-localization of TNF-α and microglial cells (c, f, i, l). The fluorescence intensity of TNF-α in the CLP group was markedly elevated compared to the sham group. Conversely, the fluorescence intensity of TNF-α was markedly attenuated in the CLP+fisetin group, but not in the CLP+placebo group compared to the CLP group. ^**^P<0.01, ^ns^P>0.05. Scale bars: 10 μm. ns: no significant.

**FIGURE S3** Fisetin downregulated iNOS in microglial cells of CLP rats. (A) The immunoreactive bands of iNOS (130 kDa) and β-actin (42 kDa). (B) The bar graph showed the upregulated expression of iNOS in the CLP group compared to the sham group. Conversely, the expression levels of iNOS were significantly downregulated in the CLP+fisetin group, but not in the CLP+placebo group compared with those in the CLP group. (C) The immunofluorescence images show the expression of iNOS (b, e, h, k, red), Iba1^+^ microglial cells (a, d, g, j, green), and the co-localization of iNOS and microglial cells (c, f, i, l). The fluorescence intensity of iNOS in the CLP group was markedly elevated compared to the sham group. Conversely, the fluorescence intensity of iNOS was markedly attenuated in the CLP+fisetin group, but not in the CLP+placebo group compared to the CLP group. ^**^P<0.01, ^ns^P>0.05. Scale bars: 10 μm. ns, no significant.
